# Association of the Genetic Polymorphisms in Pre-MicroRNAs with Risk of Ischemic Stroke in a Chinese Population

**DOI:** 10.1371/journal.pone.0117007

**Published:** 2015-02-06

**Authors:** Suli Huang, Shiquan Zhou, Yanwei Zhang, Ziquan Lv, Shanshan Li, Changhui Xie, Yuebin Ke, Pingjian Deng, Yijie Geng, Qian Zhang, Xiaofan Chu, Zhaohui Yi, Ying Zhang, Tangchun Wu, Jinquan Cheng

**Affiliations:** 1 Key Laboratory of Molecular Biology, Shenzhen Center for Disease Control and Prevention, Shenzhen, China; 2 LongHua new District Center for Disease Control and Prevention, Shenzhen, China; 3 Department of Neurology, People's Hospital of Shenzhen, Shenzhen, China; 4 Key Laboratory of Environment and Health, Ministry of Education & Ministry of Environmental Protection, and State Key Laboratory of Environmental Health (Incubating), School of Public Health, Tongji Medical College, Huazhong University of Science and Technology, Wuhan, Hubei, China; The University of Hong Kong, CHINA

## Abstract

microRNA (miRNA) plays a role in the pathogenesis of ischemic stroke, and single nucleotide polymorphisms in miRNA genes may contribute to disease susceptibility. However, the effect of miR-146a, miR-196a2, and miR-499 polymorphisms on ischemic stroke susceptibility has been rarely reported. Using the TaqMan assay, we evaluated the association of hsa-miR-146a/rs2910164, hsa-miR-196a2/rs11614913, and hsa-miR-499/rs3746444 polymorphisms with the risk of ischemic stroke in a Chinese population with 531 ischemic stroke patients and 531 control subjects. Rs2910164 C/G genotypes were significantly associated with increased risk of ischemic stroke in different genetic model (homozygote comparison: OR = 2.00, 95% CI, 1.29–3.12, *P* = 0.002; additive model: OR = 1.35, 95% CI, 1.10–1.65, *P* = 0.004;dominant model: OR = 1.33, 95% CI, 1.00–1.75, *P* = 0.049; recessive model: OR = 1.82, 95% CI, 1.20–2.74, *P* = 0.004). Subjects with allele G of hsa-miR-146a/ rs2910164 also showed increased risk of ischemic stroke (OR = 1.33, 95% CI, 1.09–1.62, *P* = 0.005). Stratification analysis showed that the association between rs2910164 and the risk of ischemic stroke was more pronounced in subjects over 60 years old, females, non-drinkers, subjects without hypertension or diabetes mellitus. There were significant combined effects between miR-146a/rs2910164 and fasting glucose/low-density lipoprotein cholesterol levels on ischemic stroke susceptibility. However, we failed to find any association between the alleles/genotypes of rs11614913 T/C and ischemic stroke, respectively (*P*> 0.05). In summary, this study provides evidence that miR-146a/rs2910164 might be associated with a significantly increased risk of ischemic stroke in a Chinese population, and the combined effects between miRNA polymorphism and fasting glucose /blood lipid levels may contribute to stroke pathogenesis.

## Introduction

Globally, stroke is the second leading cause of death for people over 60 years old [1]. In China, with 1.4 billion populations, the annual stroke mortality rate is approximately 157 per 100000, which has exceeded heart disease and become the leading cause of death and adult disability [2]. About 80% of strokes are ischemic in origin [3]. Ischemic stroke is a complex disease caused by multiple genetic and environmental factors. In addition to the conventional risk factors, such as age, sex, body mass index, hypertension, diabetes mellitus, smoking, and hyperlipidemia, single-nucleotide polymorphisms (SNPs) have been identified in genome-wide association studies (GWAS) as susceptibility loci for ischemic stroke risk [4,5]. However, such loci explain only a small portion of the total risk, and few of these SNPs discovered by GWAS involve miRNA genes.

microRNAs (miRNAs) are a class of ∼22-nucleotide non-protein coding RNAs that have emerged as key regulators of fundamental biological processes through regulating more than one third of human genes by binding to the 3’untranslated region of target gene mRNAs [6]. miRNAs are initially transcribed as primary miRNAs (pri-miRNA) with several hundred nucleotides, which are further processed into hairpin-structured precursor miRNAs (pre-miRNA) that have approximately 70 nucleotides. Pre-miRNAs are the direct precursors of mature miRNAs that have 18 to 25 nucleotides in length [7,8]. Emerging evidence supports a role of miRNAs in regulating a variety of ischemic stroke-related biologic processes, such as atherosclerosis, hypertension and plaque rupture et al [9]. miRNAs are also aberrantly expressed in ischemic stroke, and specific miRNAs have been shown to be associated with the clinical subtype of stroke and could be used as biomarker for ischemic stroke [10]. Recently, it has been proposed that the presence of genetic variants in miRNA genes could affect the processing and subsequent maturation of miRNAs [11], and collectively affect the risk and/or prognosis of diseases. Three well-known miRNA polymorphisms in pre-miRNA sequences (miR-146a C>G, rs2910164; miR-196a2 T>C, rs11614913; and miR-499 A>G, rs3746444) have been extensively studied and were found to be associated with the risk and/or prognosis of various diseases [12,13]. Interestingly, the three miRNAs also regulate genes related to thrombosis and inflammation pathways in the circulation system, including tumor necrosis factor-α (TNF-α) [14], annexin A1 (ANXA1) [15], and C-reactive protein (CRP) [16], and affect vascular damage response. However, rare data has been reported regarding the role of the miRNA polymorphisms in the pathogenesis of ischemic stroke. In this study, we sought to investigate the association between the three miRNA polymorphisms and ischemic stroke risk in a Chinese population.

## Materials and Methods

### Study population

This study is a hospital-based case-control study including 531 patients with ischemic stroke and 531 healthy unrelated volunteers. All subjects were the ethnic Han origin by self-description and unrelated Chinese people. All cases were first-diagnosed with ischemic stroke and recruited from ischemic stroke inpatients in the Department of Neurology, People's Hospital of Shenzhen (Guangdong, China) from July 2012 to July 2013. The diagnosis of ischemic stroke was based on the appearance of a new and abrupt focal neurological deficit, with neurological symptoms and signs persisting for more than 24h. Ischemic stroke was confirmed by the positive findings by head CT or MRI according to the International Classification of Disease (9th Revision, codes 430 to 438). Patients with a history of ischemic or hemorrhagic stroke, coronary heart disease, peripheral arterial occlusive disease or cancer were excluded from this study. Subjects without medical history of cerebrovascular diseases or myocardial infarction were selected as controls during a physical health examination at the hospital, and they were matched with the patients by age, sex, and area of residence. Exclusion criteria were the same as those used in the patient group, as mentioned previously. Smoking refers to people who currently or previously smoke, and drinking refers to people who currently or previously drink. Blood samples were collected and stored at −80°C until use. Genomic DNA was extracted from peripheral blood leukocyte pellets using a DNA extraction kit (AXYGEN, CA, USA).

For all participants, structured questionnaires were used to collect information on demographic characteristics and clinical biochemistry by trained interviewers. The ethics committee of Shenzhen center for disease control and prevention approved this study, and written informed consent was obtained from each participant.

### Genotyping

The peripheral venous blood sample from each individual was collected in sterile tubes with EDTA-Na2 anticoagulants and stored at −80°C. Genomic DNA of each participant was extracted from 200 μl EDTA-Na2 anticoagulated blood samples using commercially DNA isolation kit (AXYGEN, CA, USA) according to the manufacturer's instructions, and its concentration was determined by NanoDrop ND-1000 Spectrophotometer from Thermo Fisher Scientific Inc. (Waltham, MA, USA). Genotyping of the 3 SNPs was performed by TaqMan assay (Applied Biosystems) using 7500 Sequence Detection System (Applied Biosystems) in 531 ischemic stroke cases and 531 controls. Individuals who performed the genotyping were blinded to case or control status. Negative (water) control samples were included in each 96 well assay plate. About 10% of the samples were randomly selected for repeat genotyping of the three SNPs for quality control, and the results were 100% concordant.

### Statistical analysis

The normal distribution of data was tested by the One-Sample Kolmogorov-Smirnov test. The continuous variables were expressed as median (25th to 75th quartile). Differences in clinical characteristics between cases and controls were examined by the χ2 test for categorical variables and by Mann-Whitney U test for skewed parameters. All the SNPs were detected for the Hardy–Weinberg equilibrium among the controls using a χ2 test with one degree of freedom. To estimate the risk of ischemic stroke, the odds ratio (OR) and 95% confidence interval (CI) were calculated using multiple logistic regression analysis with adjustment for possible confounders, including age, sex, hypertension, diabetes mellitus, smoking, drinking and body mass index (BMI). For stratified analysis, we evaluated the gene–environment interactions by entering the multiplicative interaction term into logistic regression models, with one degree of freedom Wald test of the interaction terms. All statistical analyses were performed using SPSS 11.0 software (Statistical Package for the Social Sciences, Chicago, USA). A value of p < 0.05 was considered significant (two-tailed).

## Results

### Study population

The characteristics of patients with ischemic stroke and control subjects are summarized in **Table 1**. As expected, ischemic stroke patients are significantly more likely to have hypertension, diabetes mellitus, increased fasting glucose (FG) and low-density lipoprotein cholesterol (LDL-c) levels and decreased high-density lipoprotein cholesterol (HDL-c) level (*P*<0.05). However, total cholesterol (TC) level is significantly lower in ischemic stroke patients compared with controls (*P*<0.05). The two groups of study population show no dramatic difference in age, sex, BMI, smoking, drinking, and triglycerides level (*P*>0.05).

**Table 1:** General characteristics of the study population.

Variables	Control (n = 531)	Ischemic Stroke (n = 531)	*P* value
Male, n (%)	327 (61.6)	327 (61.6)	1.00*
Age, year	61.00 (54.00, 68.00)	63.00 (54.00, 70.00)	0.082†
BMI, kg/m^2^	23.50 (21.64, 25.63)	23.80 (22.03, 25.95)	0.103†
Smoking, n (%)	153 (28.8)	169 (31.8)	0.645*
Drinking, n (%)	100 (18.8)	101 (19.0)	1.00*
Hypertension, n (%)	115 (21.7)	340 (64.0)	<0.001*
Diabetes mellitus, n (%)	46 (8.7)	134 (25.2)	0.003*
Heart disease, n (%)	53 (10)	65 (12.2)	0.651*
FG, mmol/L	5.00 (4.60, 5.54)	5.60 (4.84, 6.99)	<0.001†
TC, mmol/L	5.20 (4.95, 5.97)	5.01 (4.33, 5.99)	<0.001†
TG, mmol/L	1.39 (1.05, 2.05)	1.46 (1.01, 2.04)	0.831†
HDL-c, mmol/L	1.10 (1.00, 1.30)	1.04 (0.87, 1.26)	<0.001†
LDL-c, mmol/L	3.00 (2.90, 3.36)	3.62 (2.81, 4.35)	<0.001†

Data are expressed as median (25^th^, 75^th^ quartiles) or percentages. BMI, body mass index; FG, fasting glucose; TC, total cholesterol; TG, triglycerides; HDL-c, high-density lipoprotein cholesterol; LDL-C, low-density lipoprotein cholesterol.

*Chi-square test for the difference in the distribution frequency between patients with ischemic stroke and control subjects.

†Mann-Whitney *U* test for the differences between patients with ischemic stroke and control subjects.

### Association of the miRNA polymorphisms with the risk of ischemic stroke

The genotype and allele frequency distributions of the three miRNA polymorphisms (rs2910164, rs11614913, and rs3746444) in ischemic stroke patients and control subjects are shown in **Table 2 and S1 Table**. The miRNA polymorphism frequencies in control subjects are consistent with the Hardy-Weinberg equilibrium expectations for rs2910164 (HWE *P* = 0.106) and rs11614913 (HWE *P* = 0.856), except for rs3746444 (HWE *P* = 0.002; as shown in **S1 Table**). Therefore, The SNP rs3746444 was excluded in further analysis. We calculated the adjusted odds ratio (OR) for rs2910164 and rs11614913 using multiple logistic regression analysis with adjustment for traditional risk factors, including age, sex, BMI, hypertension, diabetes mellitus, smoking and drinking. Results suggest that subjects with rs2910164 GG genotype had a higher ischemic stroke risk compared with subjects carrying CC genotype (OR = 2.00, 95% CI, 1.29–3.12, p = 0.002; *P*_add_ = 0.004). Rs2910164 showed a marginal association (OR = 1.33, 95% CI, 1.00–1.75, *P* = 0.049) in dominant model (CG+GG vs. CC) and significant association in a recessive model (CC+CG vs. GG, OR = 1.82, 95% CI, 1.20–2.74, *P* = 0.004). As expected, the G allele of hsa-miR-146a/ rs2910164 was also associated with significantly increased risk for ischemic stroke compared with the C allele (OR = 1.33, 95% CI, 1.09–1.62, *P* = 0.005). None of the genotype or allele of has-miR-196a2/ rs11614913 was significantly associated with the risk of ischemic stroke (*P* > 0.05).

**Table 2:** Genotype frequency of miRNA polymorphisms between ischemic stroke patients and control subjects.

Polymorphism	Control (n = 531)	Ischemic Stroke (n = 531)	OR (95% CI)*	*P* value
rs2910164				
CC	219 (41.2)	189 (35.6)	1.00 (reference)	
CG	257 (48.4)	261 (49.2)	1.19 (0.89–1.60)	0.242
GG	55 (10.4)	81 (15.3)	2.00 (1.29–3.12)	0.002
Additive model			1.35 (1.10–1.65)	0.004
Dominant model (CC vs. CG+GG)			1.33 (1.00–1.75)	0.049
Recessive model (CC+CG vs. GG)			1.82 (1.20–2.74)	0.004
C allele	695 (65.4)	639 (60.2)	1.00 (reference)	
G allele	367 (34.6)	423 (39.8)	1.33 (1.09–1.62)	0.005
HWE *P*	0.106			
rs11614913				
TT	153 (28.8)	166 (31.3)	1.00 (reference)	
TC	266 (50.1)	265 (49.9)	0.88 (0.64–1.20)	0.415
CC	112 (21.1)	100 (18.8)	0.84 (0.57–1.24)	0.367
Additive model			0.91 (0.75–1.11)	0.341
Dominant model (TT vs. TC+CC)			0.87 (0.64–1.16)	0.339
Recessive model (TT+TC vs. CC)			0.91 (0.65–1.27)	0.568
T allele	572 (53.9)	597 (56.2)	1.00 (reference)	
C allele	490 (46.1)	465 (43.8)	0.91 (0.75–1.10)	0.344
HWE *P*	0.856			

miRNA indicates microRNA; OR, adjusted odds ratio; HWE, Hardy-Weinberg equilibrium; 95% CI, 95% confidence interval.

*OR based on the risk factors, including age, sex, BMI, hypertension, diabetes mellitus, smoking and drinking.

### Stratification analysis

We performed stratified analyses according to age, sex, smoking, drinking, hypertension, and diabetes mellitus (**Table 3**). In the stratified analysis, we found that rs2910164 showed significant associations with the risk of ischemic stroke in subjects over 60 years old (OR_add_ = 1.40, 95% CI, 1.06–1.86, *P*_add_ = 0.020), females (OR_add_ = 1.65, 95% CI, 1.21–2.26, *P*_add_ = 0.002), smokers (OR_add_ = 1.56, 95% CI, 1.08–2.26, *P*_add_ = 0.018), non-smokers (OR_add_ = 1.29, 95% CI, 1.00–1.66, *P*_add_ = 0.048), non-drinkers (OR_add_ = 1.44, 95% CI, 1.15–1.82, *P*_add_ = 0.002), subjects without hypertension (OR_add_ = 1.34, 95% CI, 1.03–1.74, *P*_add_ = 0.029) and subjects without diabetes mellitus (OR_add_ = 1.30, 95% CI, 1.04–1.62, *P*_add_ = 0.019). However, no significant association was observed between has-miR-196a2/ rs11614913 genotypes and the risk of ischemic stroke in the stratification analysis. No interaction was found between the two SNPs and clinical variables, either (*P*_interaction_ >0.05).

**Table 3:** Stratification analysis of the miRNA polymorphisms.

Characteristics	rs2910164					rs11614913				
	Control*	Case*	OR_add_ (95% CI)	*P*_add_ †	*P*_interaction_	Control*	Case*	OR_add_ (95% CI)	*P*_add_ †	*P*_interaction_
Age										
< = 60	112/121/30	84/107/35	1.30 (0.95–1.76)	0.097		70/128/65	69/112/45	0.91 (0.68–1.22)	0.528	
>60	107/136/25	105/154/46	1.40 (1.06–1.86)	0.020	0.628	83/138/47	97/153/55	0.96 (0.74–1.26)	0.789	0.564
Sex										
Male	133/168/26	131/160/36	1.17 (0.89–1.54)	0.267		96/155/76	96/170/61	0.89 (0.69–1.13)	0.338	
Female	86/89/29	58/101/45	1.65 (1.21–2.26)	0.002	0.122	57/111/36	70/95/39	0.95 (0.69–1.30)	0.756	0.773
Smoking										
No	150/183/45	123/183/56	1.29 (1.00–1.66)	0.048		111/191/76	107/185/70	1.02 (0.80–1.31)	0.848	
Yes	69/74/10	66/78/25	1.56 (1.08–2.26)	0.018	0.323	42/75/36	59/80/30	0.75 (0.54–1.05)	0.093	0.133
Drinking										
No	182/203/46	145/216/69	1.44 (1.15–1.82)	0.002		123/223/85	139/213/78	0.88 (0.70–1.10)	0.235	
Yes	37/54/9	44/45/12	1.08 (0.67–1.74)	0.753	0.206	30/43/27	27/52/22	1.06 (0.69–1.62)	0.802	0.479
Hypertension										
No	172/197/47	68/91/32	1.34 (1.03–1.74)	0.029		124/206/86	58/93/40	1.01 (0.78–1.30)	0.952	
Yes	47/60/8	121/170/49	1.38 (0.98–1.93)	0.062	0.814	29/60/26	108/172/60	0.77 (0.56–1.05)	0.099	0.233
Diabetes mellitus										
No	194/238/53	138/194/65	1.30 (1.04–1.62)	0.019		142/238/105	124/198/75	0.91 (0.74–1.13)	0.393	
Yes	25/19/2	51/67/16	1.78 (0.97–3.25)	0.063	0.296	11/28/7	42/67/25	0.92 (0.54–1.58)	0.766	0.974

*Wild-type homozygote/heterozygote/variant homozygote.

†Additive model (wild-type homozygote *vs*. heterozygote *vs*. variant homozygote). Data were calculated by unconditional logistic regression, adjusted for age, sex, smoking, drinking, BMI, hypertension and diabetes mellitus.

### Combined effects between miR-146a polymorphism and fasting glucose/HDL-c/LDL-c levels on disease susceptibility

Since TG shows no significant difference between cases and control subjects, and TC level was lower in patients than control subjects, which might be due to medication use before the hospital admission of the patients, we excluded TG and TC in this study section and only analyzed the combined effects between the stroke-associated polymorphism (rs2910164) and fasting glucose, HDL-c, and LDL-c levels on the risk of ischemic stroke. Generally, high glucose, high LDL-c, or low HDL-c increases the prevalence of stroke. We divided subjects into two groups according to the median level of FG, HDL-c or LDL-c in the control subjects (5.00 mmol/L, 1.10 mmol/L, and 3.00 mmol/L, respectively). Significant combined effects were observed for rs2910164CG+GG/FG>5.00 mmol/L, and rs2910164CG+GG/LDL-c>3.00 mmol/L on the risk of ischemic stroke (**Table 4**).

**Table 4:** Combined effects of miRNA polymorphism depending on fasting glucose and lipid levels for risk of ischemic stroke.

Variables	rs2910164, OR (95% CI)*			
	Control (n)^a^	Case (n)^a^	CC	CG+GG
FG	529	508		
< = 5.00 mmol/L	112/161	60/93	1.00 (reference)	0.97(0.61–1.55)
>5.00 mmol/L	106/150	123/232	1.41(0.88–2.26)	2.32(1.52–3.54)
HDL-c	455	508		
>1.10 mmol/L	102/121	75/143	1.00 (reference)	1.93(1.23–3.01)
< = 1.10 mmol/L	89/143	110/180	1.70(1.05–2.78)	1.68(1.09–2.57)
LDL-c	384	281		
< = 3.00 mmol/L	102/143	29/60	1.00 (reference)	1.76(0.97–3.22)
>3.00 mmol/L	59/80	69/123	4.93(2.51–9.70)	4.97(2.88–8.59)

^a^ CC/CG+GG; miRNA indicates microRNA; FG, fasting glucose; HDL-c, high-density lipoprotein cholesterol; LDL-C, low-density lipoprotein cholesterol; OR, adjusted odds ratio; and 95% CI, 95% confidence interval.

*OR based on the risk factors, including age, sex, BMI, hypertension, diabetes mellitus, smoking and drinking. We divided subjects into two groups according to the median level of FG and lipids in control subjects, respectively (FG: 5.00 mmol/L; HDL-c, 1.10 mmol/L; LDL-c, 3.00 mmol/L).

## Discussion

In this study, we evaluated the associations between two miRNA polymorphisms and the risk of ischemic stroke in a Chinese population, and also estimated the gene-environment interaction and combined effects of miRNA polymorphisms and clinical characteristics. Our findings suggested that subjects carrying G allele or GG genotype of has-miR-146a/rs2910164 might have increased risk of ischemic stroke. In addition, there were combined effects for rs2910164 CG+GG genotypes and fasting glucose/LDL-c levels on the disease susceptibility.

Alteration in the expression of miRNA genes are known to contribute to the pathogenesis of stroke, including atherosclerosis, hypertension, diabetes mellitus, neuronal cell death, oxidative damage, inflammation, and edema formation [17]. miRNAs may be also novel biomarkers for cardiovascular diseases, including coronary artery disease [18], diabetes mellitus [19], stroke [10], and hypertension [20], et al. There is increasing evidence that single nucleotide polymorphisms (SNP) could make a significant contribution to disease susceptibility. However, Saunders and colleagues surveyed the publicly available SNPs data in the context of miRNA and suggested that the occurrence of SNPs in miRNA sequences is relatively rare [11], suggesting that the miRNA genes were highly conserved, which indicates that variation in miRNA might be functionally important and could influence various biological processes. Using in silico approach, Hu and collegues demonstrated for the first time that 3 SNPs with MAF>0.05 in Chinese population, were located at the pre-miRNA regions (hsa-miR-146a rs2910164 C/G, has-miR-196a2 rs11614913 C/T, and hsa-miR-499 rs3746444 A/G) [12]. Variation of the miRNA polymorphisms could affect the processing of the pre-miRNA into its mature, regulatory form, and therefore may contribute to the susceptibility to common human diseases [21]. In the present study, we found that rs2910164 was significantly associated with the risk of ischemic stroke in a Chinese population. Two recent studies reporting the associations between pre-miRNA polymorphisms and ischemic stroke susceptibility have been published during the time when we prepared for this manuscript. Consistent with our findings, Jeon et al. found that the allele G of rs2910164 was associated with ischemic stroke, while rs11614913 was not significantly different in a South Korean population [22]. In the other study, Liu and collegues analyzed three SNPs in a Chinese population (296 ischemic stroke patients and 391 healthy controls) and found that the frequency of the allele G of hsa-mir-499/rs3746444 showed significant association with ischemic stroke, while they failed to find any association between the allele/genotype of rs2910164 and rs11614913 SNPs and ischemic stroke. The discrepant findings between Liu and our study might be partially because of the difference in experimental design between the two studies. The sample size of Liu’s study is smaller, which could weaken the statistical power of the association analysis between SNP and disease risk.

The miR-146a/rs2910164, miR-196a2/rs11614913 and miR-499/rs3746444 were all located in the 3p strand of mature miRNA regions. Previous studies have demonstrated that the miR-146aG and miR-196a2T allele are associated with decreased mature miRNA levels [23,24]. In this study, we found that miR-146aG allele was associated with increased risk of ischemic stroke, inferring that decreased expression level of miR-146a might be associated with high risk of ischemic stroke. Although we did not measure miR-146a expression in the population, the literature reports show some clues to support our hypothesis. For instance, miR-146a can repress the pro-inflammatory NF-kappaB pathway as well as the MAP kinase pathway [25]. miR-146a also regulates the expression level of TNF-α [14], which is associated with the occurrence of ischemic stroke [26]. The combined effects between hsa-miR-146a/ rs2910164 and FG/LDL-c further suggested a synergetic interaction effect between miRNA polymorphism and vascular risk factors on the disease susceptibility. In addition, there might be other potential mechanisms for rs2910164 and genetic susceptibility in ischemic stroke. First, it might be some unknown target genes affecting the physiopathology of ischemic stroke. Second, the polymorphism might be in linkage disequilibrium with other genetic variations of stroke susceptibility genes located near miR-146a. However, these hypotheses need to be tested in future studies. miR-196a2 and miR-499 could regulate annexin A1[15] and CRP [16], which are also the general causes of cerebral ischemia [27,28] and associated with elevated blood pressure, BMI, insulin resistance, and triglycerides [29]. We found no association between miR-196a2/rs11614913, miR-499/rs3746444 and ischemic stroke in this study, although Liu et al reported that miR-499 G allele was significantly associated with increased risk of ischemic stroke in Chinese population [30]. These two SNPs have also been widely studied in other human diseases, including congenital heart disease [31], coronary heart disease [13] and cancer [12]. Therefore, the polymorphisms might have different effects on human diseases in specific organs, and our findings need to be validated in further studies.

There are several limitations of the present study. First, like all the case-control studies, potential selection bias could not be ruled out and might influence the interpretation of the results. Second, relatively small sample size in the present study might limit the statistical power and miss weak and potential associations, although we have considered the statistical power in this study. Since the minor allele frequency (MAF) of rs3746444 is the lowest among the three miRNA polymorphisms in Chinese population (rs11614913 MAF: 0.341; rs2910164 MAF: 0.354; rs3746444 MAF: 0.167, respectively), we calculated the statistical power using the tool Quanto according to rs3746444. This study has 88% power to detect convincing association with MAF = 0.167, α = 0.05, OR = 1.5. Further studies with larger sample size are required to confirm the role of these miRNA polymorphisms in ischemic stroke. Third, limited by the stroke subtype information, we could not analyze the role of the miRNA polymorphisms in different subtypes of ischemic stroke. Lastly, our results cannot be extrapolated to other races because interethnic variability in the frequency of stroke subtypes and genotypes may produce different results.

In conclusion, our study suggested that the miR-146a/rs2910164 C/G polymorphism might be associated with ischemic stroke risk in a Chinese population, and rs2910164 has combined effect with blood glucose and lipid on the ischemic stroke susceptibility. Further investigations in studies with larger sample size and functional tests are needed to validate our epidemiological findings and to explore the detailed biological mechanism.

## Supporting Information

S1 Table Genotype frequency of miR-499 polymorphism between ischemic stroke patients and control subjects. (DOCX)
