# Supplementary material for: Association of the Genetic Polymorphisms in Pre-MicroRNAs with Risk of Ischemic Stroke in a Chinese Population
Source: PLoS One. 2015 Feb 6;10(2):e0117007. doi: 10.1371/journal.pone.0117007 (PMC4319971; doi:10.1371/journal.pone.0117007)
Supplement: S1 Table — (DOCX) [file pone.0117007.s001.docx]

**Table S1. Genotype frequency of miR-499 polymorphism between ischemic stroke patients and control subjects**

| Polymorphism |  | Control (n=531) |  | Ischemic Stroke (n=531) |
| --- | --- | --- | --- | --- |
| miR-499 A>G (rs3746444) | | |  |  |
| AA |  | 403 (75.9) |  | 398 (75.0) |
| AG |  | 128 (24.1) |  | 133 (25.0) |
| GG |  | 0 (0) |  | 0 (0) |
| A allele |  | 934 (87.9) |  | 929 (87.5) |
| G allele |  | 128 (12.1) |  | 133 (12.5) |
| HWE *P* |  | 0.002 |  |  |

miRNA indicates microRNA; HWE, Hardy-Weinberg equilibrium.
